# Supplementary material for: Development of an HPLC-MS/MS Method for the Determination of Silybin in Human Plasma, Urine and Breast Tissue
Source: Molecules. 2020 Jun 24;25(12):2918. doi: 10.3390/molecules25122918 (PMC7356828; doi:10.3390/molecules25122918)
Supplement: Supplementary file 1 [file molecules-25-02918-s001.pdf]

molecules

Article

Development of an HPLC-MS/MS method for the determination of silybin in human plasma, urine and breast tissue.

Matteo Lazzeroni[1†]; Giovanna Petrangolini[2†*], José Antonio Legarreta Iriberri[3], Jaume Pascual Avellana[3], Digna Tost Robusté[3], Sara Cagnacci[1], Debora Macis[1], Valentina Aristarco[1],Bernardo Bonanni[1], Paolo Morazzoni[2], Harriet Johansson[1§] and Antonella Riva[2§]

[1]Division of Cancer Prevention and Genetics, IEO-European Institute of Oncology, IRCCS, Via Ripamonti 435, 20141 Milan, Italy.

[2]Research and Development Department, Indena S.p.A., Viale Ortles 12, 20139 Milan, Italy.

[3]Bioanalysis Department, Kymos Pharma Services S.L., Ronda Can Fatjó 7-B, 08290 Cerdanyola del Vallès, Spain.

* Correspondence: Corresponding author: Matteo Lazzeroni, MD. Division of Cancer Prevention and Genetics. IEO-European Institute of Oncology, Via Ripamonti 435, 20141 Milan, Italy. matteo.lazzeroni@ieo.it

† These authors contributed equally to the study.

§ These authors contributed equally to the study as senior scientists.

(a)

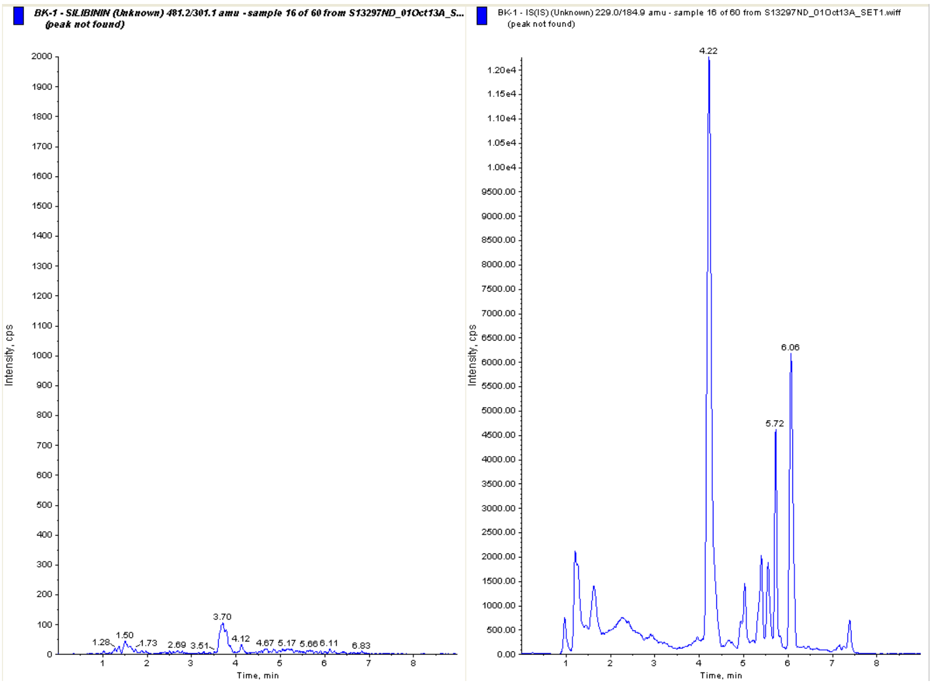

(b)

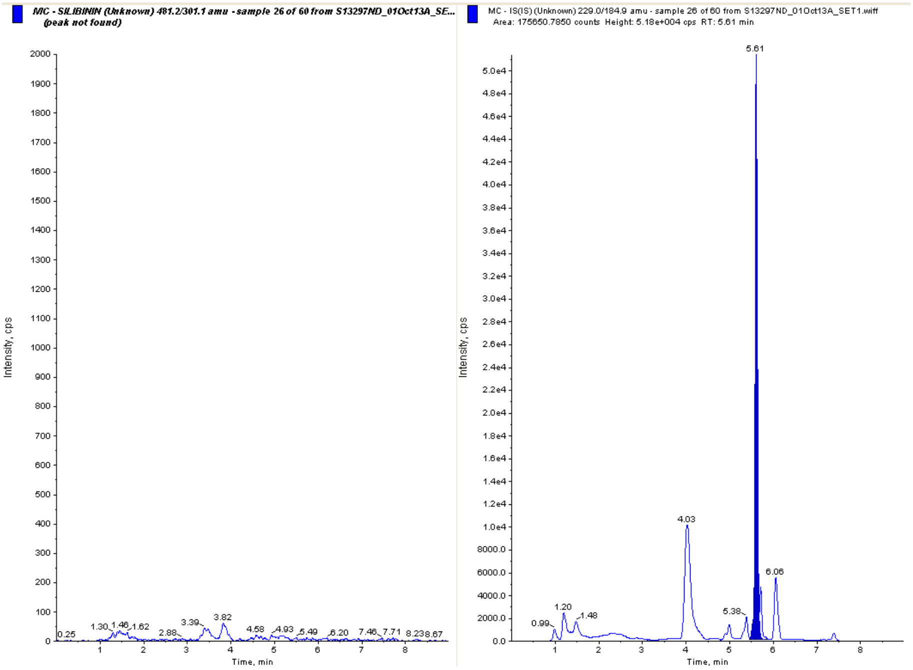

Supplementary Figure 1. MRM chromatogram corresponding to a urine blank sample (Panel A) and a urine zero sample (Panel B). In each panel: on the left MRM chromatogram corresponding to silibinin, on the right MRM chromatogram corresponding to the internal standard.

(a)

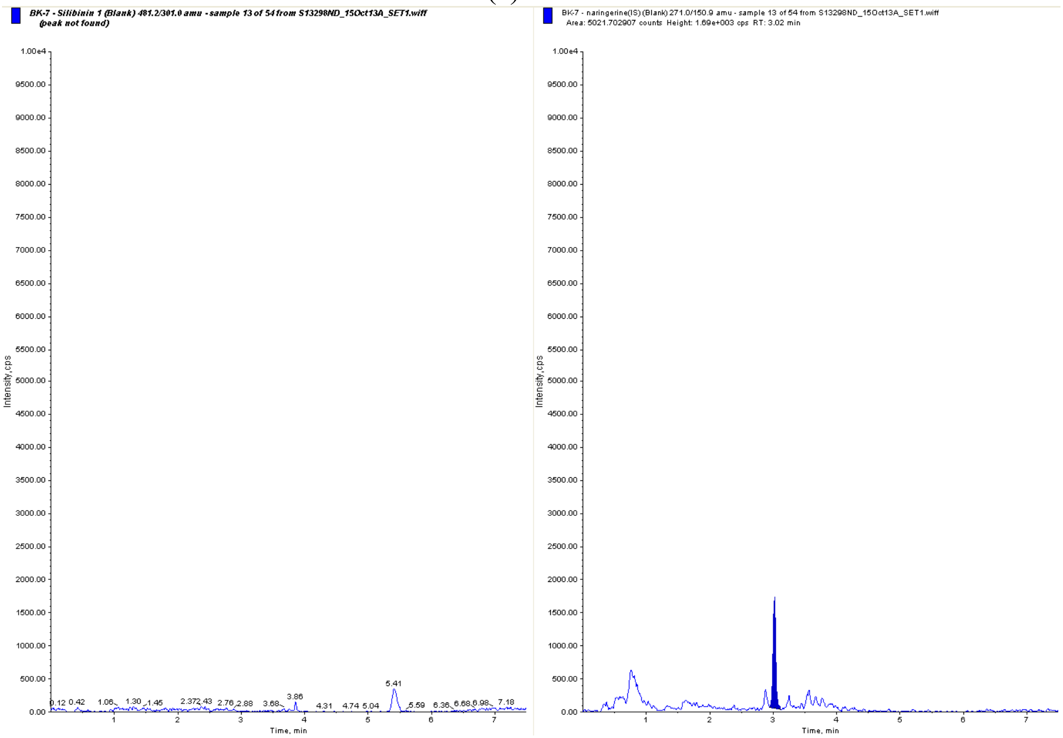

(b)

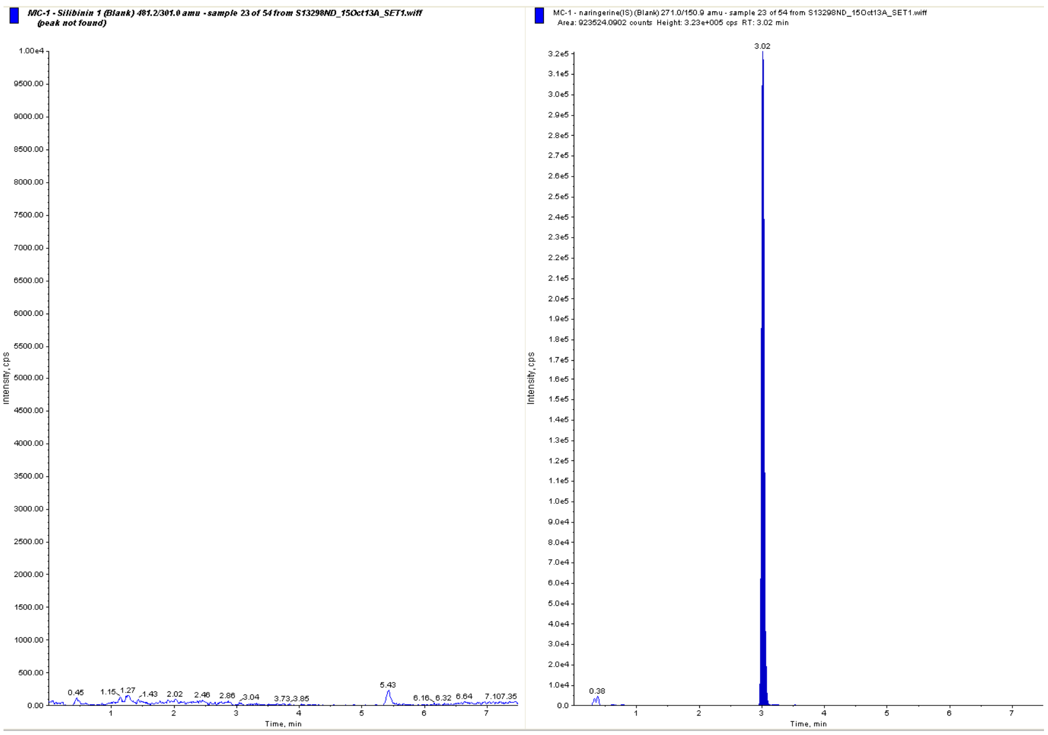

Supplementary Figure 2. MRM chromatogram corresponding to a pig muscle blank sample (Panel A) and a pig muscle zero sample (Panel B). In each panel: on the left MRM chromatogram corresponding to silibinin, on the right MRM chromatogram corresponding to the internal standard.

Supplementary Table 1. Total and free Silybin levels in plasma, urine, breast cancer, and adjacent unaffected breast tissue in patients who received silybin for 28 days [17].

		Median	Q1	Q3	Min	Max	P-value
**TOT-SIL (plasma) ng/mL**	Baseline	0	0	0	0	0	
	Before last administration	901	651	1481	214	4574	
	2 hrs after last administration	14538	13147	16828	7654	31121	0.0004¥
**SIL (plasma) ng/mL**	Baseline	0	0	0	0	0	
	Before last administration	69	13	159	1	523	
	2 hrs after last administration	5847	4526	6454	1818	10861	0.0004¥
**TOT-SIL (urine) ng/mL***	Baseline	0	0	0	0	0	
	At surgery	7131	2015	21095	90	26573	
**SIL (urine) ng/mL***	Baseline	0	0	0	0	0	
	At surgery	212	71	359	7	747	
**TOT-SIL (tissue) ng/g**	Tumor	131	35	869	0	1375	
	Normal breast tissue	11	0	34	0	48	0.018
**SIL (tissue) ng/g**	Tumor	33	4	158	0	177	0.013
	Normal breast tissue	0	0	3.79	0	19	

Twelve breast cancer patients received silybin-phosphatidylcholine, 2.8 g daily for 4 weeks prior to surgery. Silybin levels were measured before (SIL) and after (TOT-SIL) enzymatic hydrolysis by high-performance liquid chromatography (HPLC)-MS/MS in biologic samples (plasma, urine, breast cancer, and surrounding normal tissue). Fasting blood samples were taken at baseline, before the last administration, and 2 hours later. All patients were fully compliant and completed the treatment program.

*: creatinine normalization; end: before the last administration; end-2h: 2 hours after the last administration.

¥ P for the difference between Before last administration and 2 hrs after last administration.
